# Condylar Changes after Maxillary Expansion in Children with Cleft Lip and Palate—A Three-Dimensional Retrospective Study

**DOI:** 10.3390/biomimetics7020073

**Published:** 2022-06-05

**Authors:** Inês Carolina Graça, Inês Francisco, Adriana Guimarães, Francisco Caramelo, Francisco Vale

**Affiliations:** 1 Institute of Orthodontics, Faculty of Medicine, University of Coimbra, 3000-075 Coimbra, Portugal; ines.carolina.may@gmail.com (I.C.G.); ines70.francisco@gmail.com (I.F.); adrianarguima@gmail.com (A.G.); 2 Coimbra Institute for Clinical and Biomedical Research (iCBR), Area of Environment Genetics and Oncobiology (CIMAGO), Faculty of Medicine, University of Coimbra, 3000-075 Coimbra, Portugal; fcaramelo@fmed.uc.pt; 3 Laboratory of Biostatistics and Medical Informatics (LBIM), Faculty of Medicine, University of Coimbra, 3004-531 Coimbra, Portugal; 4 Centre for Innovative Biomedicine and Biotechnology (CIBB), University of Coimbra, 3000-075 Coimbra, Portugal

**Keywords:** cleft palate, cone beam computed tomography, mandibular condyle, malocclusion, palatal expansion technique

## Abstract

Background: The presence of posterior crossbite can trigger aesthetic and functional changes as mandibular asymmetry in individuals, contributing to asymmetrical muscle function. Mandibular asymmetry and respective condyle adaptation may be an etiological factor in temporomandibular disorder. This study aims to evaluate the effects of maxillary expansion on the position and angulation of the condyles as well as the intercondylar distance in children with cleft lip and palate. Methods: Twenty-five individuals with cleft lip and palate who underwent maxillary expansion were selected. Condylar changes were evaluated by cone beam computed tomography using the Pullinger and Hollender formula. To determine the statistically significant differences between the variables, the Student t-test and the Benjamini–Hochberg correction method for multiple comparisons were used. Results: No statistically significant differences between angulation and condylar position before and after maxillary expansion were found. The intercondylar distance tended to increase in growing individuals with cleft lip and palate after maxillary expansion. Conclusions: Intercondylar distance shows a tendency to increase after expansion regardless of the cleft phenotype. No differences were found in angulation and condylar position with the changes in occlusion resulting from maxillary expansion.

## 1. Introduction

The cleft lip and palate (CLP) are the most common congenital malformations of the craniofacial region, with an incidence of 1 per 700 live births. Prevalence can be affected by sex, race, ethnic groups, geographical regions, and socioeconomic conditions [1,2,3]. The etiology of CLP is multifactorial, involving genetic and environmental factors, namely smoking consumption and ionizing radiation [2,4,5,6]. Regarding genetic factors, some of the best-supported genes and genetic loci are the following: IRF6, MAFB, ARHGAP29, ch8q24, ventral anterior homeobox (VAX1) and paired box protein Pax-7 (PAX7). Some cleft syndromes have been associated with a genetic locus, namely FGFR2 in Crouzon syndrome, TCOF1 in Treacher Collins syndrome, IRF6 in Van der Woude syndrome and SOX9 in Pierre Robin syndrome [7]. Recently, some gene-environment interactions have been suggested as factors that increase the risk of cleft, such as MTHFR polymorphisms and maternal folate intake, alcohol and ADHIC variants and NATI 1095 polymorphism and lack of maternal multivitamins [8,9,10]. The main complaints of CLP patients are functional and aesthetic, affecting mainly mastication, hearing, phonation, dentofacial development and the correct function of the upper airways [2,3].

Palate repair is associated with three major complications: oronasal fistula, velopharyngeal insufficiency and alteration of the pattern of craniofacial growth [11]. An oronasal fistula may persist if normal palatal wound healing fails, which occurs more frequently for larger defects, tissues under tension, or local infection. Therefore, an oronasal communication will persist, which may lead to regurgitation of fluids between the oral and nasal cavity, abnormal speech, facial deformity, malocclusion, and psychological impairments [12,13].

The distinct pattern of craniofacial growth among CLP individuals in the general population is mainly due to lip and scar tissue resulting from surgical interventions on the lip and/or primary palate, which may induce posterior displacement of the maxilla [14]. Some studies suggested that the cleft can modify the mandibular spatial position since the cleft promotes a downward and backward rotation of the mandible associated with a vertical growth pattern [7]. Additionally, dental anomalies are significantly more frequent in CLP, which can promote the development of malocclusions [1,15,16]. The most common malocclusions observed are: skeletal class III due to maxillary retrognathia, crowding and anterior and posterior crossbite [6,15,17]. The posterior crossbite is also one of the most common malocclusions during deciduous, mixed and permanent dentition [18]. The presence of this malocclusion, unilateral or bilateral, can trigger dental and functional changes, namely at the level of muscle activity fatigue, contributing to asymmetrical muscle function [2]. The consequent neuromuscular adaptation, acquired by the position of the mandible, can lead to asymmetrical mandibular growth, facial disharmony and skeletal crossbite in permanent dentition [18]. Mandibular asymmetry and corresponding condyle adaptation may be an etiological factor in temporomandibular disorder (TMD) since it can affect temporomandibular joint (TMJ) morphology [1,3,18,19]. This adaptive capacity promotes interpersonal variations that result from the growth of the condyles and the ascending mandibular ramus in several directions [3]. Thus, the existence of malocclusion or a continuous anomalous muscle function can promote a process of remodeling of the TMJ [1,3]. Consequently, the position of the condyle, the ascending ramus, and the shape of the mandibular fossa can change, which may lead to a predisposition to TMD. However, it is noteworthy that TMD has a multifactorial etiology. In order to avoid these consequences, the transversal maxillary discrepancy must be treated as soon as possible [18,20]. Maxillary expansion is commonly used as part of a sequential treatment for cleft patients since it can correct the transversal constriction caused by previous surgeries, establish the maxillary arch, and expansion appliances can be used to stabilize the upper arch during a secondary bone graft [17,18,21]. The evaluation of possible condylar asymmetries as a consequence of posterior crossbite and asymmetric muscle function can be studied using three-dimensional imaging such as cone beam computed tomography (CBCT). CBCT has a greater definition than conventional two-dimensional methods and less radiation exposure when compared to computed tomography. Additionally, the American Academy of Oral and Maxillofacial Radiology provides several clinical recommendations on the use of CBCT in CLP care, since these patients have medical conditions that require adequate 3D analysis for an accurate diagnosis [22].

Knowledge on the influence of orthodontic treatment in TMJ improves treatment standard of care since it helps to identify the functional impacts, contributing to the planning of clinical interventions. This is even more important in patients with cleft lip and palate due to the burden of care associated with the long and multidisciplinary treatments that they perform. During the last years, the clinical changes of maxillary expansion on TMJ have been reviewed, but studies on cleft patients are sparse. Therefore, this study aims to assess the effects of maxillary expansion on the position and angulation of the mandibular condyle, as well as the mandibular fossa in CLP patients through CBCT evaluation.

## 2. Materials and Methods

This retrospective study was approved by the Ethics Committee of the Faculty of Medicine of University of Coimbra (CE-144/2020—according to the 1964 Helsinki declaration and its later amendments or comparable ethical standards). All participants gave their written informed consent prior to the start of the study. 

The sample was selected according to the following inclusion criteria: CLP individuals; patients undergoing maxillary expansion; patients with a complete medical history; CBCT and dental casts before and after maxillary expansion. The exclusion criteria were: the presence of syndromes; previous orthognathic surgery (except surgery to close the primary lip and palate); degenerative TMJ diseases; condylar fractures; individuals with incomplete data or an assessment of the structures was not possible; and patients with atypical clefts.

**Figure 1:**
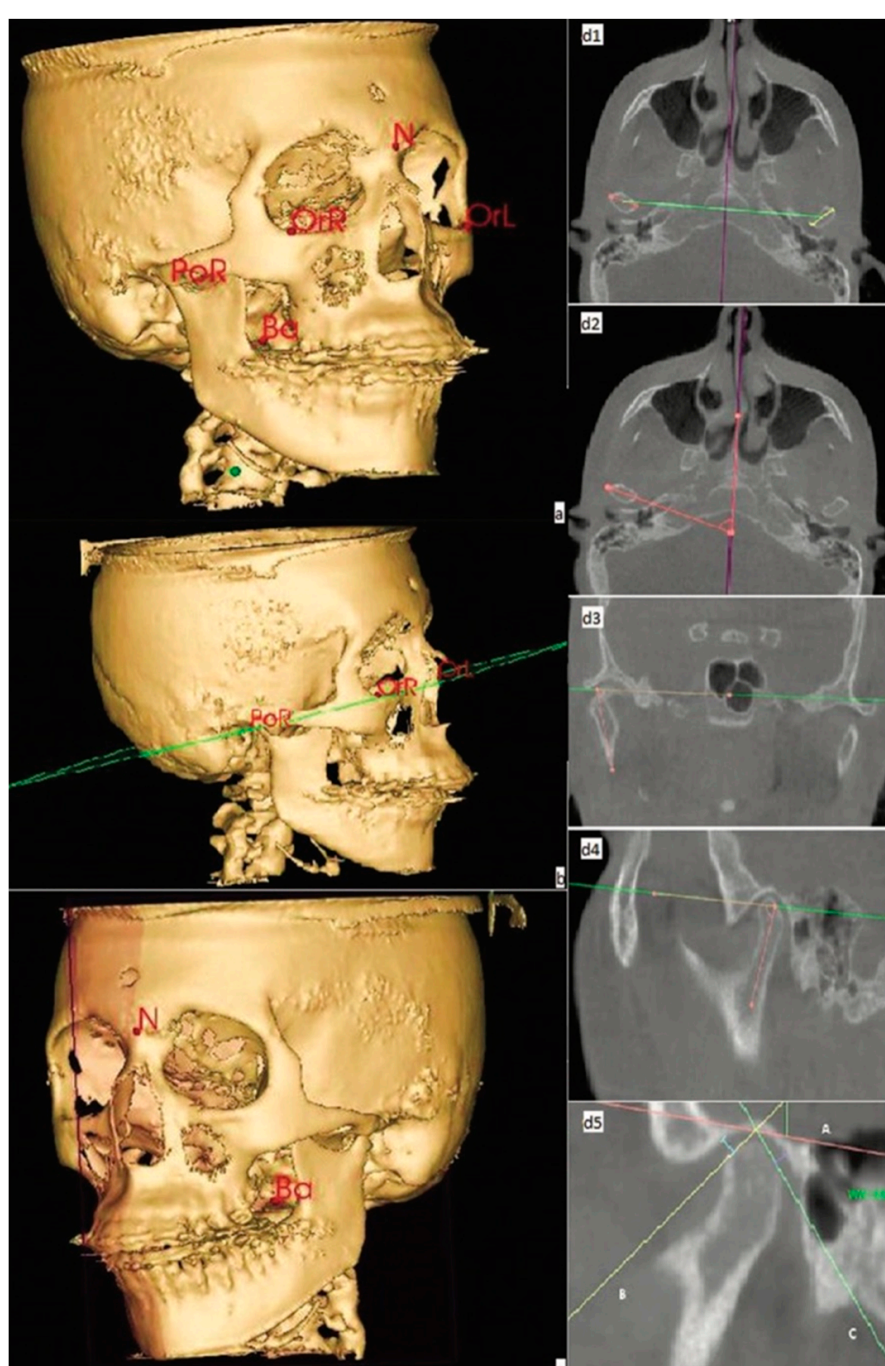
CBCT image evaluation: (**a**) midpoints of reference; (**b**) Frankfort horizontal plane; (**c**) midsagittal plane; (**d1**) intercondylar distance; (**d2**) condylar angulation in the axial plane; (**d3**) condylar angulation in the frontal plane; (**d4**) condylar angulation in the sagittal plane; (**d5**) condylar position in the sagittal plane. Adapted from Vale et al. [23].

CBCT images were collected by a technician trained prior to the study to assess the mandibular condyle and mandibular fossa at two points of evaluation: T0, before maxillary expansion; and T1, after maxillary expansion. The parameters used to perform the CBCT were: 120 kVp, 5 mA, 4 s scan time with an axial layer thickness of 1 mm, 16 × 10 cm field of view and 0.30 mm^3^ voxel size. CBCT images were obtained with a natural position of the head, maximum intercuspation and the tongue and lips in a resting position. The images were then converted into the DICOM (Digital Imaging and Communications in Medicine) format and the data were analyzed using 3D-OS Nemoceph software (Software Nemotec SL, Madrid, Spain). The following variables were evaluated: condylar angulation (measured in axial, frontal and sagittal planes), the intercondylar distance (measured in the axial plane) and the condylar position (measured in the sagittal plane). In order to carry out the necessary measures, the average reference points and guide plans were marked as illustrated in Figure 1.

Subsequently, the variables were measured according to the methods defined by Vale et al. [23]. All measurements were performed by a single authors follows:

1. Condylar angulation. (a) Axial plane: determined by the angle between the line that connects the medial and lateral pole of the condyle head and the medial sagittal plane. (b) Frontal plane: determined by the angle formed by the axis of the ascending mandibular ramus and the horizontal plane of Frankfort (PHF). (c) Sagittal plane: determined by the angle formed at the bisection of the anterior and posterior margin of the condyle head and the PHF.

2. Intercondylar distance. Determined by the distance between the midpoint of the right condyle and the midpoint of the left condyle.

3. Condyle position. The condyle position in the mandibular fossa is determined in the sagittal plane, where a line is drawn (A-line) formed from the uppermost point of the mandibular fossa and parallel to the PHF. From the A-line, two tangents are performed on the anterior and posterior margins of the condyle head (called B- and C-lines). Subsequently, using a line perpendicular to these tangents, the anterior and posterior distance to the condyle was measured.

The position of the condyle in the glenoid fossa was determined by using the Pullinger and Hollender formula (Table 1).

The statistical analysis was performed using the IBM^®^ SPSS^®^ v26 statistical platform (SPSS, Inc., Chicago, IL, USA) and R v3.3.2. Descriptive statistics included the following variables: mean, standard deviation, minimum and maximum value. The difference between the two time points was evaluated using the Student’s *t*-test for paired samples after verifying an assumption of normality with the Shapiro–Wilk test. Given the high number of comparisons, the Benjamini–Hochberg correction method for multiple comparisons was chosen, adopting a value of 5% for the false positive rate (false discovery rate). Angular and condylar position values are presented by radar charts, and the intercondylar distance is displayed in a scatter plot. To assess the agreement between the two time points, the iota coefficient [24] was used and a Bland–Altman graphic was created for some of the tested measures. A significance level of 0.05 was adopted.

## 3. Results

The selected sample comprised twenty-five patients with CLP who underwent maxillary expansion. The sample included patients between 8 and 24 years old, with a mean age of 15.9 years and a male/female ratio of 16/9. Regarding the type of the cleft, ten individuals had a left unilateral CLP, six had a right unilateral CLP, seven had a bilateral CLP, one had a secondary palate cleft, and one had a soft palate cleft. All patients used a quad helix as an expander device. 

None of the variables evaluated presented statistically significant differences (Table 2). The left and right frontal condylar angles appeared to increase and were statistically significant according to Student’s test (0.030 and 0.031, respectively); however, after applying the Benjamini–Hochberg correction method, the *p*-value increased to 0.114. All angular values tended to increase after expansion, except the right condylar angle in the sagittal plane, which decreased from 72.83° to 71.31°. In contrast, the contralateral angle increased from 69.63° to 71.26°. 

Regarding condyle position, the right condyle showed a posterior inferior movement after expansion and the left condyle performed an anterior inferior movement. Despite this discrepancy, both condylar angles became more homogeneous in the sagittal plane. In the axial plane, there were only small variations after expansion with both condyles showing a slight medial rotation. It was also verified that the right and left condyles on average remained, before and after expansion, in an “anterior” position in the mandibular fossa.

**Figure 2:**
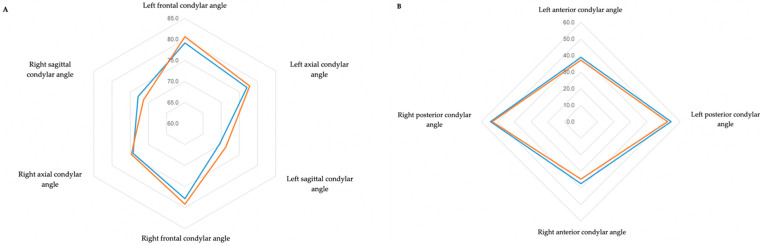
(**A**) Radar chart of condylar angles in the three planes of space. (**B**) Radar chart of condylar angles in the sagittal plane to assess condylar position.

The angulation in the frontal plane increased on average from 79.17° to 80.62° for the left condyle and 77.87° to 79.17° for the right condyle. Moreover, both condyles performed a medial rotation movement. The angles and position of the condyles are shown in Table 2 and Figure 2, respectively. It was found that, on average, there was a decrease in all angles after maxillary expansion. The anterior angle on the right side showed the greatest decrease (37.30° to 34.67°), and its posterior angle showed the smallest variation (54.50° to 53.33°). 

**Figure 3:**
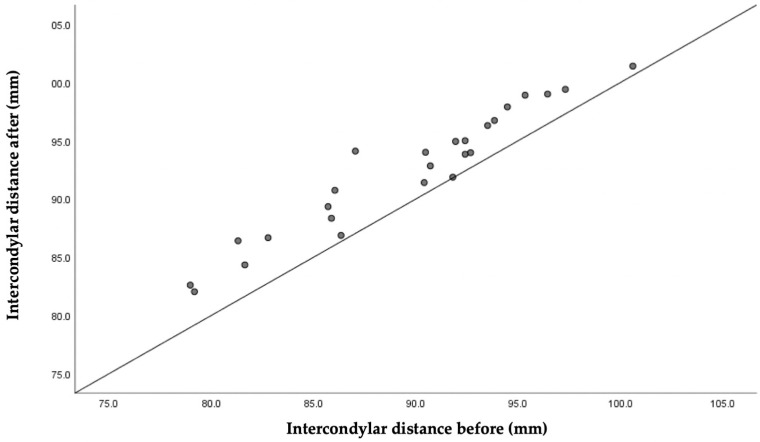
Relationship between the intercondylar distance before and after expansion.

Concerning the intercondylar distance, the relationship between the two points of evaluation is presented in Table 3 and Figure 3. Most of the points in Figure 3 are above the y = x line, which shows that the intercondylar distance tended to increase after maxillary expansion. The change in intercondylar distance was statistically significant since both tests yielded a *p*-value less than 0.001.

**Figure 4:**
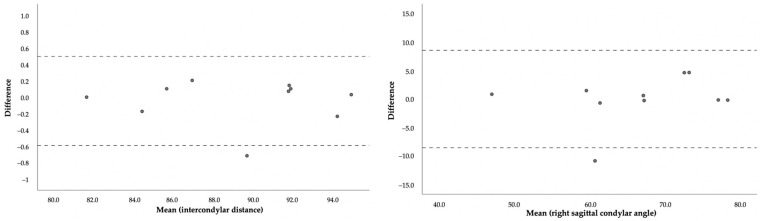
Bland–Altman plots to assess agreement between time points of evaluation.

The iota coefficient for the agreement was equal to 0.885, showing a high intra-observer agreement. The Bland–Altman charts used to assess agreement are presented in Figure 4.

## 4. Discussion

Knowledge about the position and angulation of the mandibular condyle is crucial for understanding craniofacial development in individuals since the condyles are a growth site and therefore play an important role in mandibular development [25]. Differential condyle growth can be caused by asymmetric masticatory muscle activity due to occlusal problems [26]. The present study aimed to evaluate changes in the position and angulation of the mandibular condyle, as well as the mandibular fossa, in CLP patients after maxillary expansion. It was verified that regardless of the CLP phenotype, the intercondylar distance tended to increase, and most patients maintained an anterior condylar position in the mandibular fossa after maxillary expansion.

Posterior crossbite is often found on the cleft side in patients with CLP. This malocclusion can trigger continuous abnormal muscle function that can promote a TMJ remodeling process. In this study, it was found that the positions of the left and right condyles in the mandibular fossa before treatment did not show specific differences. This finding is in accordance with a study by Leonardi et al. [27], which verified that the position of the condyles in the mandibular fossa were maintained in healthy patients that underwent maxillary expansion. The symmetry between the condylar positions before performing maxillary expansion can be explained by compensatory remodeling of the condyle, which may or may not be associated with variation in the thickness of the TMJ articular disc [27]. Similarly, no condylar asymmetry was found before expansion in this study. On the other hand, the correction of posterior crossbite was performed at an early age, which contributed to the elimination of asymmetric muscle activity. Kiliaridis et al. found that patients who underwent orthodontic treatment do not manifest asymmetrical muscle activity [28].

The selected sample underwent maxillary expansion using a quad helix appliance. This appliance allows basal and dentoalveolar expansion through a slow expansion protocol, allowing physiological adaptation of the tissues. A more physiological adaptation of tissues is extremely important in individuals with CLP, since recurrent cleft closure surgeries promote the formation of fibrous scar tissue. As previously mentioned, occlusal alterations triggered by transverse expansion promote TMJ alterations and this was verified in this study, since an increase in the intercondylar distance was observed, regardless of the CLP phenotype. These results are in agreement with Ghoussoub et al. [29], which found a significant transversal increase in the intercondylar distance in growing patients without CLP after rapid maxillary expansion using the Hyrax appliance. Additionally, some reports [29,30] have shown that maxillary expansion with the quad helix appliance does not cause significant changes in the condylar position in relation to the mandibular fossa. Holberg et al. reported that bilateral and unilateral CLP patients experience greater skeletal expansion with the use of the quad-helix appliance than patients without CLP [31]. Additionally, it was found that the anterior and posterior angles of the condyles decreased with no statistical significance. Although the difference between final and initial values, both at an angular and a positional level, was small (1.3° on the right side and 1.45° on the left side in the frontal plane), the change at the clinical level was greater. Thus, this study emphasizes the need for early treatment of posterior crossbite in order to reestablish normal craniofacial growth and functional balance and avoid the development of mandibular and condylar asymmetries and TMD. Moreover, the occlusal changes caused by expansion do not promote significant variations in the position and angulation of the condyles in the mandibular fossa, which reinforces the safety of this orthodontic therapy.

Although this study verified that maxillary expansion can be performed with relative safety, there are some limitations that have to be discussed. First, the comparison of this outcome with other studies is difficult since studies with CLP patients are scarce and there are methodological differences between studies. Second, the sample size did not allow subgroup analysis. Third, it is difficult to differentiate whether the increase in the intercondylar distance resulted from the physiological changes of maturation or from maxillary expansion. Be that as it may, this study has several strengths, namely, the use of CBCT to assess the condyles in the three planes of space (instead of conventional two-dimensional methods) as well as a high intra-observer agreement.

Future studies should include a control group of non-CLP individuals who undergo maxillary expansion in order to understand how the presence of this congenital malformation can affect the results. Additionally, a larger sample would allow the comparison of different CLP phenotypes.

## 5. Conclusions

Intercondylar distance shows a tendency to increase after expansion regardless of the cleft phenotype. No differences have been found in angulation and condylar position with the changes in occlusion resulting from maxillary expansion.

## Figures and Tables

**Table 1:** Condylar position classification according to Pullinger and Hollender.

Measure	Description
ln (PS/AS) > 0.25	Anterior position of the condyle in the glenoid fossa
ln (PS/AS) < 0.25	Posterior position of the condyle in the glenoid fossa
−0.25 < ln (PS/AS) < 0.25	Concentric position of the condyle in the glenoid fossa

AS, anterior space; P, posterior space.

**Table 2:** Condylar angle and position before and after maxillary expansion. The values are presented in the following order: mean (standard deviation) minimum/maximum.

Variable (Plane)		Initial (°)	Final (°)	*p* ^§^	*p* ^£^
Condylar angle	Left (frontal)	79.17 (4.07)	80.62 (4.02)	0.030	0.114
		70.43/88.70	73.93/88.80		
	Left (axial)	77.08 (6.35)	77.81 (5.72)	0.527	0.644
		59.30/88.63	65.10/86.37		
	Left (sagittal)	69.63 (8.77)	71.26 (7.49)	0.379	0.580
		49.43/84.90	58.17/85.23		
	Right (frontal)	77.87 (3.79)	79.17 (4.00)	0.031	0.114
		70.37/84.97	73.03/88.33		
	Right (axial)	74.18 (5.70)	74.70 (6.22)	0.637	0.698
		61.73/86.87	64.07/88.73		
	Right (sagittal)	72.83 (5.71)	71.31 (8.43)	0.370	0.580
		61.03/84.00	47.43/85.10		
Condylar position	Left anterior angle	38.80 (12.12)	37.12 (10.67)	0.422	0.580
		13.57/65.27	10.77/65.00		
	Left posterior angle	54.41 (12.11)	52.27 (11.00)	0.416	0.580
		28.93/70.43	35.53/72.70		
	Right anterior angle	37.30 (10.32)	34.67 (11.21)	0.270	0.580
		19.30/56.00	9.23/60.33		
	Right posterior angle	54.50 (13.71)	53.33 (10.68)	0.698	0.698
		31.60/80.27	30.37/71.20		

^§^ Student *t*-test. ^£^ Benjamin–Hochberg.

**Table 3:** Intercondylar distance before and after maxillary expansion. The values are presented in the following order: mean (standard deviation) minimum/maximum.

Variable	Initial (°)	Final (°)	*p* ^§^	*p* ^£^
Intercondylar distance	89.56 (5.83) 78.97/100.60	92.35 (5.41) 82.03/101.40	<0.001	<0.001

^§^ Student *t*-test. ^£^ Benjamini–Hochberg.

## Data Availability

The data presented in this study are available on request from the corresponding author.
